# Age-associated bidirectional modulation of gene expression in single identified R15 neuron of *Aplysia*

**DOI:** 10.1186/1471-2164-14-880

**Published:** 2013-12-14

**Authors:** Beena M Kadakkuzha, Komolitdin Akhmedov, Tom R Capo, Anthony C Carvalloza, Mohammad Fallahi, Sathyanarayanan V Puthanveettil

**Affiliations:** 1 Department of Neuroscience, Scripps Florida, 130 Scripps Way, Jupiter, FL 33458, USA; 2 Information Technology and Informatics, The Scripps Research Institute, Scripps Florida, 130 Scripps Way, Jupiter, FL 33458, USA; 3 Division of Marine Biology and Fisheries, University of Miami Rosenstiel School of Marine and Atmospheric Science, 4600 Rickenbacker Causeway, Miami, FL 33149, USA

**Keywords:** *Aplysia californica*, Bi-directional gene regulation, Single neuron transcriptome, R15 neuron, Neuronal circuitry, Signaling networks, Aging, CREB, S6 Kinase

## Abstract

**Background:**

Despite the advances in our understanding of aging-associated behavioral decline, relatively little is known about how aging affects neural circuits that regulate specific behaviors, particularly the expression of genes in specific neural circuits during aging. We have addressed this by exploring a peptidergic neuron R15, an identified neuron of the marine snail *Aplysia californica.* R15 is implicated in reproduction and osmoregulation and responds to neurotransmitters such as acetylcholine, serotonin and glutamate and is characterized by its action potential bursts.

**Results:**

We examined changes in gene expression in R15 neurons during aging by microarray analyses of RNAs from two different age groups, mature and old animals. Specifically we find that 1083 ESTs are differentially regulated in mature and old R15 neurons. Bioinformatics analyses of these genes have identified specific biological pathways that are up or downregulated in mature and old neurons. Comparison with human signaling networks using pathway analyses have identified three major networks [(1) cell signaling, cell morphology, and skeletal muscular system development (2) cell death and survival, cellular function maintenance and embryonic development and (3) neurological diseases, developmental and hereditary disorders] altered in old R15 neurons. Furthermore, qPCR analysis of single R15 neurons to quantify expression levels of candidate regulators involved in transcription (CREB1) and translation (S6K) showed that aging is associated with a decrease in expression of these regulators, and similar analysis in three other neurons (L7, L11 and R2) showed that gene expression change during aging could be bidirectional.

**Conclusions:**

We find that aging is associated with bidirectional changes in gene expression. Detailed bioinformatics analyses and human homolog searches have identified specific biological processes and human-relevant signaling pathways in R15 that are affected during aging. Evaluation of gene expression changes in different neurons suggests specific transcriptomic signature of single neurons during aging.

## Background

Aging is a ubiquitous process that involves the progressive deterioration of physiological functions. Many of the behavioral changes associated with aging in humans are also observed in lower organisms. These changes include, but are not limited to, a decline in learning and memory, locomotor ability, olfactory sensitivity, and circadian rhythmicity [1,2]. Since behavior is largely affected by the functional state of the nervous system, age-related behavioral declines could very likely indicate the perturbations in the brain. As an organism ages, most tissues undergo multilayered changes and are subject to cellular damage that accumulate with age. It has been well established that there are changes in gene expression in both model organisms and humans as they age. DNA microarray studies on whole worms and flies have also been used to profile transcriptional changes of aging [3-5]. Large-scale transcriptional changes in mice and primates identified a number of functional genes that change expression levels during aging [6,7].

Understanding the gene expression changes that occur during aging in the human brain is of particular interest due to its relation to both normal and pathological neurodegeneration and is particularly challenging due to the inherent complexity of the brain at the anatomical and cellular level. Like other biological systems, the complexities of nervous systems at different levels have been studied using a variety of animal models [8-13]. These animal models have been instrumental with the identification of key neuronal functions in humans and have demonstrated conserved signaling pathways that are critical in aging and neurodegenerative diseases [14-18].

Fundamental brain functions are performed by subsets of neurons – neuronal circuits – that are classically defined as distinct networks of hundreds of neurons. The development of neural circuits is initiated with synaptic connections and refined by systematic neuronal activity. Due to the close relationship between neural connectivity and neural activity throughout the brain, it is essential to consider how neural circuitry function at the single neuronal level. Recent studies suggest that experience can dictate the number of neurons or circuits regulating a specific behavior [19,20]. These studies emphasize the significance of probing gene functions at the circuit and single neuron level.

To understand aging at the single neuron level, we explored the marine snail *Aplysia californica* that are characterized by a simple central nervous system (CNS). *Aplysia* CNS has been extensively studied to understand molecular and physiological changes during memory storage and neural circuitries underlying specific behaviors. For example, Kandel and colleagues identified neural circuits underlying the gill withdrawal reflex response and have identified several key determinants of learning and memory storage [21,22]. Studies by Peretz and coworkers have described the age dependent sensitivity of the gill withdrawal reflex and osmoregulation [23-26]. A key advantage of using *Aplysia* is that specific behavior is mediated by a simple circuitry [21,22,27-29]. Moreover the changes associated with a specific behavior can be mapped back to the level of single neurons, thus providing access to molecular and biochemical mechanisms of basic neuronal functions at single-neuron resolution. This possibility offer one of the biggest advantages of employing *Aplysia* for studying molecular and cellular basis of behavior when compared to other model organisms such as worms, flies and mice. Exploring these advantages, age-associated changes in gene expression of identified neurons R2 and LPl5 from abdominal ganglia of young (3–4 months) and old (7–10 months) animals were recently described [30]. Significant changes in gene expression in old R2 neurons, and a unique set of genes differentially expressed during aging were identified [31].

Senescence in *Aplysia* is characterized by declining weight, reproduction (egg laying), reflex response and finally death in 11–13 months [32]. Impairment of the long-term retention of habituation, prevention of acquisition of sensitization in the gill withdrawal reflex (GWR) and reduction of the response to food stimuli [33-35] are associated with aging in *Aplysia*. At the macromolecular level aging associated changes include transcriptional and translational changes [31] in response to circadian rhythm [31], and the change in electrophysiological properties in identified neurons [34].

Despite these studies the details of how aging alters gene expression and specific signaling pathways in single neurons and circuitries remains to be determined. To address this, we have focused on age-associated gene expression changes in *Aplysia* neuron R15. The R15 neuron is one of the best-studied neurons used to understand single neuron burst dynamics [36-39], neuronal circuitry functions and cellular basis of behavior [40]. R15 has been a subject of extensive electrophysiological studies due to its role in egg laying [41-43], neurochemical modulation [44,45].

Using custom designed microarrays [36] we compared the gene-expression profiles of single R15 neurons from mature (6 months; reproductively mature) and old (11–12 months; close to senescence) animals and identified several genes that are bidirectionally regulated. Our selection of the specific ages (6 month group and 11–12 months group) for this study is based on the fact that previous studies in *Aplysia* demonstrated robust changes in behavioral readouts and electrophysiological properties occurring between 4–6 months when the animal is attaining sexual maturity [2,24,35], and the behavioral and physiological expressions start to get weakened in animals 250 days (8.5 months) of age and older [37]. Therefore we assumed that by selecting ages 6 month and 11–12 months, we would be able to capture the significant differences in gene expression and signaling pathways. Next, we mapped these differentially expressed genes to human functional pathways and identified three networks involved in cell death, neurological diseases and cell morphology. Finally, using single neuron qPCR analyses, we found that aging alters expression of key transcription regulators (CREB1, CREB2) and translation modulator (S6 kinase) in identified neurons R15, L7, L11 and R2 in unique ways.

## Results

### Identification of differentially expressed genes during aging of single identified neuron R15

Earlier studies have shown that *Aplysia* exhibit behavioral changes associated with aging [33]. We assumed that age-dependent changes in specific behaviors in *Aplysia* are associated with specific molecular changes in single neurons. To investigate age associated molecular changes in single neurons, we studied R15 neurons isolated from two age groups that are six months apart, sexually mature (6 months old from hatching) and old (11–12 months from hatching). *Aplysia* reach senescence in laboratory conditions by 12–14 months [38,39]. As described in the “Background” section, R15 has been subjected to extensive electrophysiological characterization to gain insights into the biophysical mechanisms underlying neuron endogenous bursting [45-47], and is implicated in reproduction, and osmoregulation [41-43,48].

**Figure 1:**
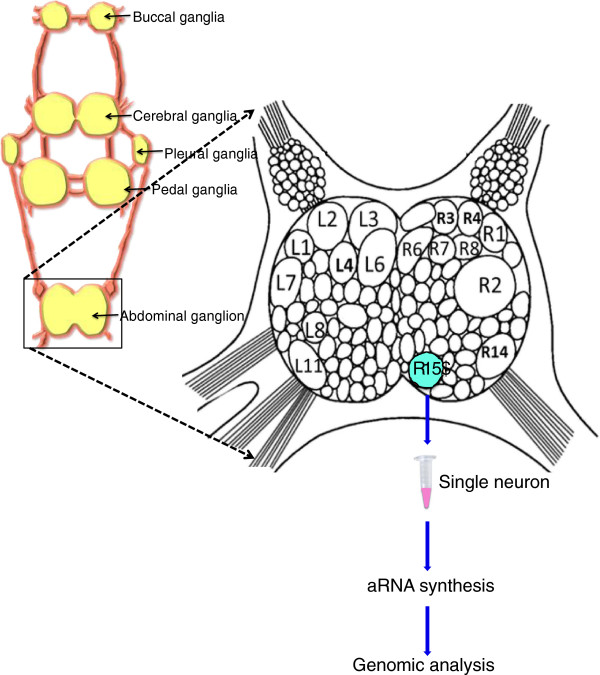
**Preparation of R15 single neurons from *Aplysia *abdominal ganglia for genomic analysis. ***Aplysia* central nervous system is organized into nine different ganglia. Identified neurons of abdominal ganglia are shown. Single R15 neuron was isolated from anesthetized *Aplysia* and immediately stored in Trizol at -80°C. Total RNA was isolated using standard Trizol RNA isolation method and amplified using MessageAmp™ II aRNA Amplification Kit. Quality control and quantification of freshly isolated CNS was performed using a Bioanalyzer and by spectrophotometry.

The overall strategy employed to identify differentially expressed genes is shown in Figure 1. To identify age-associated changes, we followed an unbiased gene expression analysis using custom designed microarray that contain a 44,000-oligonucleotide array (60-mer oligonucleotide sequences; Agilent Technologies) that were designed from each non-redundant sequence in the *Aplysia* EST database [36,49].

**Figure 2:**
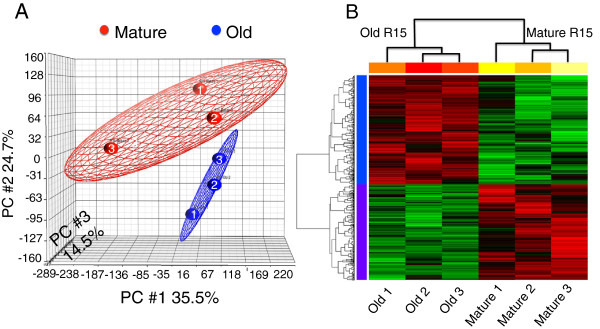
**Heat map analysis of microarray data showing differentially expressed genes between mature and old single R15 neurons. (A)** Principle Component Analysis (PCA) mapped scatter plot: The global gene expression profiles of the R15 neurons from both mature and old *Aplysia* were analyzed by PCA. The figure represents the first two principal components of microarray analysis data (PC1, PC2 and PC3) in X, Y and Z respectively, and demonstrated the expression profile of the three single neurons per group (Mature Red # 1–3, Old blue# 1–3). **(B)** Heat map analysis of microarray data showing hierarchical clustering of 1083 differentially expressed probes between R15 mature and old single neurons. Each group has three single neurons tested. Red or green colors indicate differentially up or downregulated genes, respectively. The mean signals were background corrected and transformed to the log2 scale. Genes with at least 2-fold changes with p < 0.05 at the 95% confidence level were considered as significant.

Single R15 neurons were isolated from abdominal ganglia of mature and old animals, each group in biological triplicates. We carried out dual color microarray analyses following linear amplification of total RNAs from individual neurons. Scatter plots and correlation coefficients for each single neuron were calculated (Additional file 1: Figure S2A). The correlation between the biological replicates in each group was calculated using principle component analysis (PCA) mapping as shown in Figure 2A. Figure 2A shows the first three principal components of microarray analysis data (PC1, PC2 and PC3) in X, Y and Z respectively, and demonstrate correlation between the expression profile of the three single neurons per group (Mature Red # 1–3, Old blue# 1–3). Similarly the PCA was calculated for the technical replicates for each neuron and the first three principal components (PC1, PC2 and PC3) in X, Y and Z respectively showed a positive correlation (Additional file 1: Figure S2B). Our analysis of microarray data have identified 1083 probe sets that are differentially expressed based on the criteria of a fold change of ≥2 and adjusted p < 0.05 between mature versus old neurons (Figure 2B).

The 1083 differentially expressed probes (Additional file 2: Table S1) were annotated using a new *Aplysia* sequence resource database generated by the Institute for Genome Sciences (IGS), at the University of Maryland. The sequences were generated by The Broad Institute and were distributed to the community (http://www.aplysiagenetools.org) through the Institute of Genome Sciences (IGS), University of Maryland. After considering a 2-fold change cut-off, we identified 572 differentially expressed transcripts, 247 were enriched in R15 mature and 325 were enriched in R15 old neurons. This corresponded to ~56% upregulated transcripts and ~43% downregulated transcripts during aging. The top 9 transcripts that are enriched in mature or old are listed in Table 1. The complete list of differentially expressed transcripts in mature and old are listed in Additional file 2: Table S2. The differentially expressed genes include, kinases (MAP kinase), phosphatases (phosphoglycolate phosphatase), ion channels (C9 serotonin receptor), neuropeptides (L11), transcription factors (CREB2) protein synthesis regulators (S6K), and those involved in regulating metabolism (adenylate cyclase).

**Table 1:** Age associated differentially expressed genes in mature and old R15 single neurons

**Microarray probe**	**Accession ID**	**Aplysia genes**	**Fold change**		**E-value**
**ID**			**Mature**	**Old**	
UF_Ac_120286_a1	NM_001204602.1	p38 MAP kinase	9		4.00E-111
UF_Ac_100654_b3	NM_001204622.1	Carboxy	6		1.00E-45
		peptidase D			
UF_Ac_107934_m1	NM_001204626.1	PKA type II regulatory subunit	4		5.00E-06
UF_Ac_100564_b3	NM_001204701.1	CREB2	3		0
CUST_1610_PI418051350	NM_001204704.1	S6 kinase	2		1.00E-13
CUST_2099_PI418051350	NM_001204691.1	Churchill		2	2.00E-112
UF_Ac_120932_a1	NM_001204726.1	Adenylate cyclase		3	2.00E-18
UF_Ac_105136_m1	M14958.1	FMRFamide		3	2.00E-54
UF_Ac_100549_b3	NM_001204616.1	Temptin		19	0

### GO analysis of differentially expressed genes in R15

**Figure 3:**
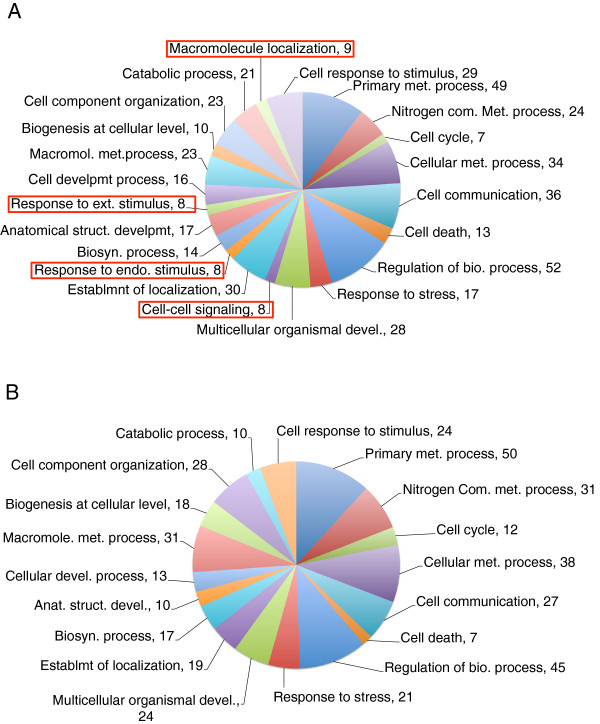
**Functional annotation of differentially expressed genes based on Gene Ontology (GO) classification.** Pie chart representing numbers of upregulated **(A)** from a total of 178 gene IDs and downregulated **(B)** from a total of 181 gene IDs in corresponding Gene Ontology Biological Process layer 2 categories in the mature R15 single neuron. Biological processes that are uniquely regulated are labeled using a red rectangle. Numbers show the unique hits corresponding to a biological process category.

To understand the biological significance of the microarray data the annotated *Aplysia* sequences were mapped to gene ontology (GO) biological process category. We identified several GO categories specific among genes upregulated or downregulated with age (Figure 3A and B). We found 178 genes upregulated in the mature neurons that were mapped into 22 biological process categories at level three of the GO analysis. However, there are only 18 GO categories for 181 genes that are downregulated in the mature neurons (Additional file 2: Table S3).

Four GO categories (cell-cell signaling (GO:0008219), macromolecule localization (GO:0043170), response to endogenous stimulus (GO:0051234), and response to external stimulus (GO:0051716) are unique to the upregulated gene data set of mature neurons (Figure 3A). With the exception of a few processes, the number of sequences upregulated or downregulated in each GO category is roughly similar, indicating a bidirectional differential expression during aging.

Further analysis of the key functions of specific genes belonging to a particular GO category and the directionality of the expression suggest their possible role in altered biological functions associated with aging. For example, in the category “catabolic process” (GO:0007049) enriched sequences are significantly higher than the downregulated sequences. One of the sequences, kinesin 1b, is downregulated with aging. Kinesin is a motor protein involved in the transport of cargos such as synaptic vesicles and mitochondria from cellbody to synapses and is a critical mediator of long-term memory storage. On the other hand, eukaryotic translation initiation factor 2-alpha kinase 3 that is involved in the repression of global protein synthesis, is upregulated with age as its expression is higher in the neurons of old *Aplysia*. Key regulators of signaling cascades such as MAP kinase 14 and MAP kinase-activated protein kinase 2, and transcription factor 4 were consistently downregulated in R15 with age. Intriguingly, adenylate cyclase and Churchill were upregulated in R15 with age. Adenylate cyclase is involved in cyclic AMP metabolism whereas Churchill is involved in neural development.

### Identification of human homologs of Aplysia genes that are altered during aging

**Figure 4:**
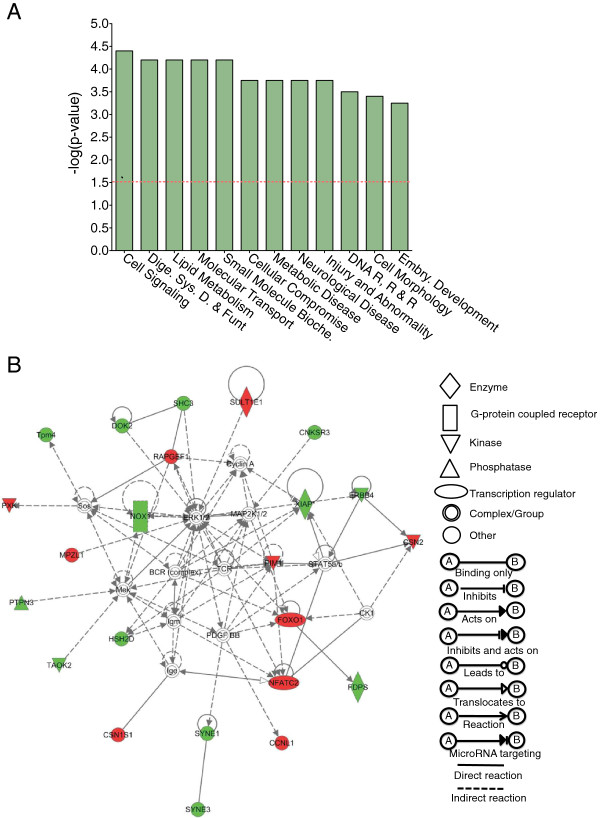
**Mapping of differentially expressed genes into human pathways.** Differentially expressed transcripts showing a +/- 2-fold change or greater were selected from the microarray data were analyzed using IPA software to identify potential homologous human pathways. From the 572 gene IDs of the differentially expressed gene set between R15 mature and old single neurons, 190 human homologs were identified. **(A)** Top 12 pathways from IPA analysis **(B)** Network 1, identified from IPA analysis, has 25 genes differentially expressed that are associated with cell signaling, cell morphology, and skeletal muscular system development. Node (gene) and edge (gene relationship) symbols are shown along with network diagram. Red shows genes that are upregulated and green indicates down regulation of the genes. Uncolored notes represent genes that were not identified in our data set as differentially expressed in our analysis, but relevant to this network.

Next, we searched for human homologs of genes that are bidirectionally regulated in R15 during aging. Of the 572 gene IDs from the differentially expressed gene set between mature and old R15 neurons, 190 IDs were successfully mapped into different human pathways using Ingenuity Pathway Analysis (IPA). The top 12 functional categories that met stringent statistical criteria and significant representation of genes are illustrated in Figure 4A. The mapped datasets contain either upregulated or downregulated genes associated with specific functional pathways (Additional file 2: Table S4).

We next focused on the top three signaling networks from the IPA analysis. Network 1 (Figure 4B) has 25 genes differentially expressed and associated with cell signaling, cell morphology, and skeletal muscular system development. FOXO1, (a transcription factor of the Fox family) one of the upregulated genes in the network, has been implicated in aging process [50,51]. Network 2 has 25 genes differentially expressed that are involved in cell death and survival, cellular function maintenance and embryonic development (Additional file 1: Figure S3A). Two genes in Network 2 ATR (Ataxia Telangiectasia and Rad3 related) and MLH1 (MutL Homolog 1) are upregulated in the mature neuron and earlier studies showed their role in aging and longevity [52,53]. Network 3 has 15 genes differentially expressed and is associated with neurological diseases, developmental and hereditary disorders (Additional file 1: Figure S3B). Thus our mapping of *Aplysia* single neuron gene expression signatures into the human database has identified several evolutionarily conserved genes that are important for human aging.

### Aging of R15 is associated with specific changes in expression of CREB and S6 kinase genes

**Figure 5:**
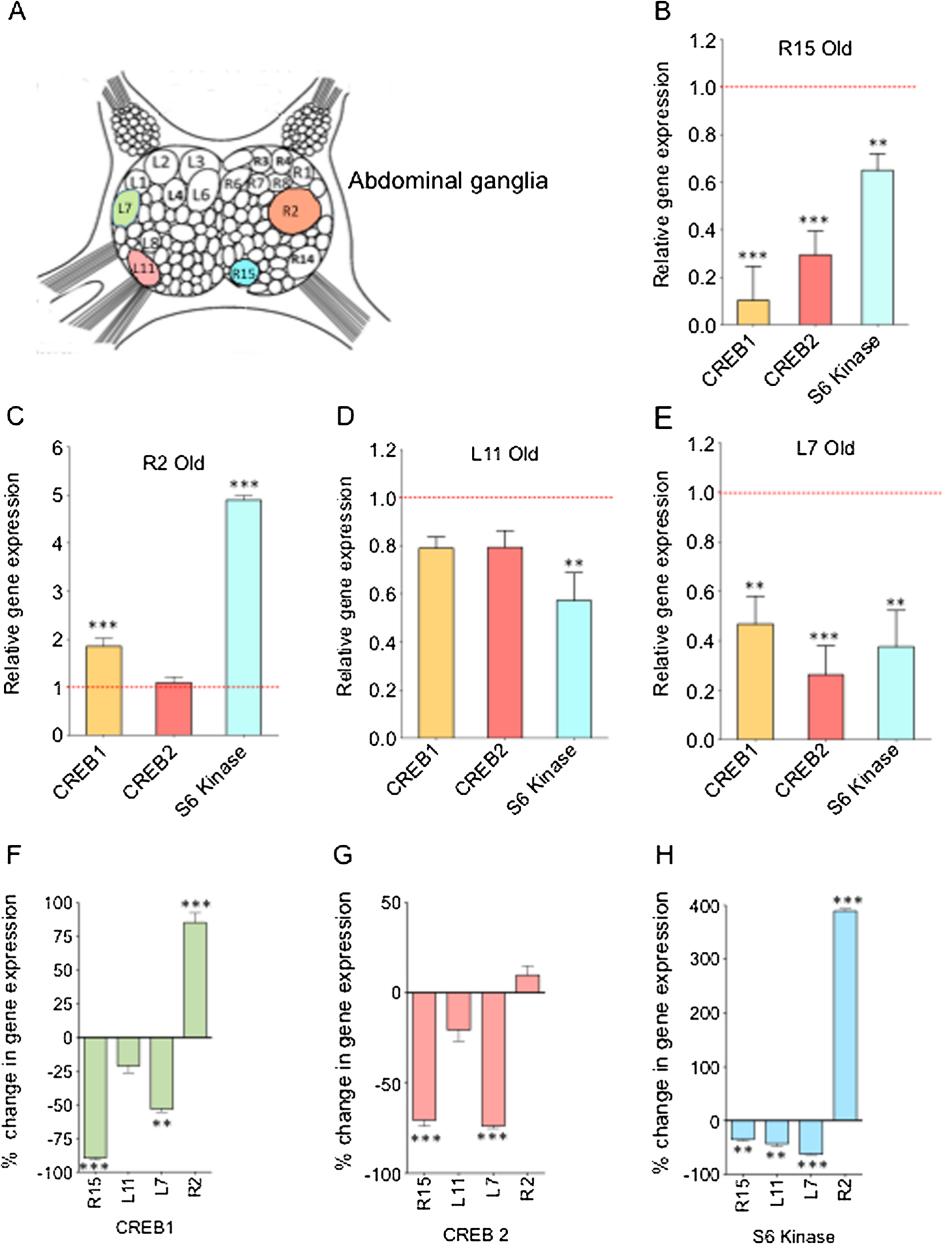
**Aging related changes in the expression of CREB1, CREB2 and S6K in identified single neurons of the abdominal ganglia.** Single neuron qPCR analysis of expression of CREB1, CREB2 and S6K mRNAs in old neurons when compared to that of mature neurons are shown in bar graphs. **(A)** Identified neurons in the abdominal ganglia of *Aplysia*. Single neurons analyzed in this study are color labeled; **(B)** In the case of old R15, all three genes are significantly downregulated; **(C)** In old R2, CREB1 and S6K are significantly upregulated. CREB2 did not change with aging in R2; **(D)** In old L11, CREB1 and CREB2 do not show any significant down regulation, whereas S6K is downregulated significantly; **(E)** In old L7, all the three genes are downregulated significantly. Figures 5F-H represent the percent change in gene expression of individual genes in different identified neurons of abdominal ganglia during aging. **(F)** Significant changes in expression of CREB1 in R15 (90 ± 5%), R2 (80 ± 5%) and L7 (50 ± 5%). The change in L11 was not significant; **(G)** CREB2 showed significant changes in expression levels in R15 (70 ± 5%) and L7 (70 ± 5%) and but no significant changes in R2 and L11; **(H)** S6K expression changes significantly in all four neurons, R15 (30 ± 5%), L11 (50 ± 5%), L11 (70 ± 5%), and R2 (400 ± 15%). Ct values of target genes were normalized to 18S rRNA internal standard. Each measurement consists of 5 biological repeats, 4 technical repeats and the error bars indicate standard error of the mean (SEM). Student’s t test determined statistical significance where **P-value < 0.01, ***P-value < 0.001.

Transcription and translation are two molecular processes that undergo dynamic changes during aging process [54-58]. To quantitate changes in transcription and translation in the R15 neuron during aging, we carried out quantitative PCR (qPCR) analysis of expression of CREB1 and CREB 2 (transcription factors) and S6 kinase (S6K, a regulator of translation) in mature and old abdominal ganglia (Additional file 1: Figure S1) and individual neurons. Importantly, these genes are identified as downregulated genes in old R15 neuron from our microarray analysis. We find from qPCR analysis that CREB1 in mature and old ganglia did not show any significant differences in expression (Additional file 1: Figure S1) whereas the single neuron analysis showed significant changes in expression during aging. Specifically, CREB1 showed significant change in the expression levels with 10 fold decrease in the old neuron, where as CREB 2 is ~ 3 fold and S6K is ~1.3 fold downregulated in the old neuron (Figure 5B) confirming our microarray data. Student t test was used to evaluate the statistical significance while calculating the fold change between mature and old single neurons and a *p*-value < 0.05 is considered statistically significant.

Next we examined whether CREB and S6K are downregulated during aging of other neurons in the abdominal ganglia. To address this question we studied gene expression in three identified neurons, R2, L7 and L11 (Figure 5A). L7 and L11 are two motor neurons and R2 is a cholinergic neuron. L7 is well characterized for its role in learning and memory of gill withdrawal reflex [16,59,60]. Using qPCR we quantified the CREB1, CREB2 and S6K transcript levels in mature and old neurons. In contrast to R15, mRNAs for CREB1 (~2 fold) and S6K (~5 fold) were upregulated significantly whereas CREB2 mRNA was not changed (Figure 5C) in R2. Interestingly, we found that in old L11 neuron, CREB1 and CREB2 mRNAs were not changing significantly, however S6K mRNA was ~1.5 fold downregulated (Figure 5D) whereas in L7 neurons, CREB1, CREB2 and S6K mRNAs were downregulated significantly (~2 fold, ~3 fold and ~2 fold respectively; Student’s t test; p < 0.05) (Figure 5E).

### Aging induced changes in gene expression are neuron specific

To further understand the neuron-specific changes during aging, from single neuron qPCR measurements we calculated the percentage changes in the expression of each transcript in different neurons. We observed 80 ± 10% changes in the expression of CREB1 mRNA in R15 (downregulated) and R2 (upregulated) neurons whereas in L7, CREB1 expression was 50 ± 5% downregulated. Unlike significant changes in CREB1 mRNA levels in R15, R2 and L7 the expression of CREB1 mRNA did not significantly change in L11 single neuron (Figure 5F) during aging. Similarly, we measured the percentage change in CREB2 mRNA levels and found that in R2 and L11, the CREB2 mRNA levels were not affected by aging, however there were significant changes in R15 (60 ± 10%) and L7 (65 ± 10%) (Figure 5G). Interestingly, S6K mRNA expression was affected by aging in all neurons. However the magnitude of change varied significantly among these four neurons. In the R15 and L11 neurons S6K mRNA expression changed 35 ± 10% (downregulated in both) whereas in L7 neuron the percentage change was 60 ± 10% (downregulated). Change in R2 neuron was very different from the rest of the neurons with 400 ± 15% upregulation (Figure 5H).

## Discussion

Aging is a fundamental biological process that has distinct phenotypical, physiological and molecular level characteristics. The gene expression level change hypothesis of aging is one of the principal theories of age-associated changes in cellular functions in many tissues, including the brain [30,56,61]. Accordingly, the functional genomics approach is one of the useful ways for describing some of the molecular characteristics of neuronal changes underlying normal aging and neurodegeneration [30,62-65]. Considering the cellular heterogeneity of the brain and that behavior is regulated by specific neural circuitries, single circuit or single neuron expression analysis could be a better way to look into the specific molecular changes associated with behavioral decline during aging. Such studies might provide novel therapeutic targets and accelerate future drug discovery efforts. Our comparative analysis of CREB1 expression during aging in the intact ganglia and individual neurons highlight the importance of single neuron study of aging. While we did not find any statistically significant differences in the expression of CREB1 in the abdominal ganglia (Figure 1) during aging, we observed significant differences in CREB1 expression in our single neuron measurements. In this study, we have explored the age-associated molecular changes in four different identified neurons (R15, R2, L7 and L11) in the abdominal ganglia of *Aplysia*.

### Bidirectional changes in gene expression during aging

Our microarray analyses of gene expression in single R15 neurons have identified a subset of genes, 572 of which are bi-directionally regulated during aging. We studied two age groups, sexually mature (6 months) and old animals that are close to senescence (11–12 months). Of the 572 genes we found that 247 genes were upregulated and 325 genes were downregulated in mature R15 neurons when compared to old R15 neurons. Based on the conservative estimate that *Aplysia* contain approximately 20,000 genes and that our custom oligonucleotide array correspond to approximately 11,000 genes, we estimate that this array contain about 50-60% expected transcriptome. Therefore the change in the gene expression of 1083 transcripts suggests that approximately 10% of the genes are bi-directionally regulated (≥ 2 fold change, p-value ≤ 0.05) in the R15 neuron during aging. However this change might not reflect the net changes in transcriptome during aging. The changes only reflect mRNA regulation in the two age groups we studied. It is quite possible that noncoding transcriptome might be regulated differently.

In our study, differentially expressed genes from both age groups fall into several functional categories that include transcription, translation, protein synthesis, degradation and metabolism. This alteration of gene expression identified in the R15 neuron is consistent with the molecular aging data from other organisms ranging from fly to human where a considerable number of genes that belong to different functional categories undergo dynamic gene expression during aging [66]. Our finding that specific sets of genes that are upregulated or downregulated in single old neurons, indicate that age-associated changes are bidirectional at the molecular level. The age-linked decline in many physiological processes are in fact associated with an increase in the expression of many genes that could alter specific signaling pathways by generating an imbalance in the functional networks, hence affecting key biological functions. Consistent with this idea, the GO category analysis of microarray data in Figure 3 shows that most of the differentially expressed genes share common GO. GO terms corresponding to cell-cell communication, response to internal stimulus, response to external stimulus, and macromolecule localization was absent in old neurons. These biological processes are vital for the maintenance of neuronal health and we find that during aging these processes become defective. Thus our studies suggest significant signaling imbalance associated with aging.

### Regulation of human relevant signaling pathways in R15 during aging

We next analyzed our data in the context of human aging by mapping on to human pathways using Ingenuity Pathway Analysis (IPA). Differentially expressed genes were further mapped to identify signaling pathways homologous to human. 190 human genes were mapped in the IPA gene list marking ~35% of annotated genes differentially expressed in *Aplysia*. The top three pathways are associated with cell death and survival, cellular function and maintenance, cell signaling and neurological disease. Further supporting our notion that aging is associated with an imbalance in signaling, we find that both upregulated and downregulated genes were mapped to the same network. Thus the up or down regulation of nodes of the networks will affect the output of the network, affecting various biological processes. At the single cell level, few key biological processes that are affected by aging include intra and inter-cellular transport, cell-cell communications, transcriptional and translational regulation and epigenetic regulations, all of which significantly change the functioning profile of a cell. Therefore these results are likely to be relevant in the context of human aging.

### Transcription and translation are differentially regulated in single neurons during aging

Transcription and translation are fundamental to cell maintenance and survival. CREB has been studied extensively in the context of brain specific functions such as synaptic plasticity [67-69], long-term memory [70] and aging [71,72]. Recently CREB has been shown to be involved in human aging [73], cognitive and neurodegenerative disorders [74]. In mice, CREB regulate aging related genes such as the A-T-mutated gene (ATM) [72]. Consistent with these observations, we find that the expression of CREB is altered at the single neuron level during aging.

Ribosomal S6 kinase (S6K), a translation regulator, is differentially expressed in R15 during aging. S6K is a downstream target of rapamycin (mTOR) pathway, a key modulator of aging [75]. S6K is involved in several signaling pathways [76,77] that contribute to various pathological states, including aging-related pathology [78,79] and extending the life span of mammalian cells [79]. In agreement with these results obtained for mammalian aging, we find from our single neuron microarray and qPCR analysis that S6K is downregulated during aging of *Aplysia* R15 neuron.

### Differential aging of neural circuitries

Moroz and Kohn (2010) have shown, using gene expression analysis, that two cholinergic neurons, LPI1 and R2, age differentially [30]. We studied CREB1, CREB2 and S6K mRNA expression in four neurons (R15, L7, L11 and R2). In support of the differential aging idea, we find that age-dependent changes in expression of these genes are neuron specific. We made two conclusions from the gene expression analysis of these four identified neurons. First, we find that during the aging process expression of genes are differentially affected in neurons. In R15 and L7 neurons, the CREB1, CREB2 and S6K showed significant decrease in expression, whereas in L11 only expression of S6K was affected. In R2, unlike the other three neurons during aging, CREB1 and S6K were upregulated. Second, the extent to which expression of each gene is altered depends on the neuron. For example, CREB1 showed ~85% decrease in expression in R15 whereas L7 showed a 50% decrease. On the other hand, the percentage decrease in CREB2 in R15 and L7 were not significantly different. Interestingly, the CREB2 levels did not change in old L11 and R2. Since these neurons are part of circuitries involved in different behaviors, our results suggest that as in the case of single neurons, different neural circuitries could also age differently and may directly contribute to the specific neuronal correlates of aging. Consistent to this idea, it was shown that different regions of mammalian brain age differently and that changes in specific circuitries are associated with behavioral decline during normal aging [80-82].

## Conclusions

Our study demonstrates the importance of analysis of single neurons to understand transcriptome changes associated with aging. We conclude from our single neuron analysis that genes undergo changes in expression levels during aging. These changes could be bidirectional- sets of genes upregulated and sets of genes downregulated during aging. Importantly, several molecular changes during aging of mammalian cells are identified in aging *Aplysia* neurons suggesting that molecular changes associated with aging involve several conserved mechanisms. Furthermore, all neurons do not necessarily undergo similar changes in gene expression. For example, in the case of the R2 neuron, transcriptional and translational regulators that we studied were upregulated during aging whereas expressions of these regulators were downregulated in the other three neurons. Also the magnitude of change in expressions of genes is specific to individual neurons. This now poses a question - how and why aging affects neurons differently? Whole transcriptome and epigenome analyses of identified neurons might address this challenge.

## Methods

The Institutional Biosafety Committee of The Scripps Research Institute (TSRI) approved all the experimental protocols (IBC Protocol 2010-019R1) described in this study. There are no ethical approvals required for the research using invertebrate animals, such as *Aplysia,* which are not included in the list of regulated animals. However, we have discussed the experiments with the Institutional Animal Care and Use Committee of TSRI and every effort was made to minimize suffering of *Aplysia*.

### Single neuron isolation and RNA analysis

Mature (6 months) and old (11–12 months) *Aplysia* were obtained from the NIH/University of Miami National Resource for *Aplysia* and maintained at 16°C under 12:12 light–dark conditions in the Instant Ocean artificial sea water (ASW). Methodology to isolate single neurons for RNA analysis was recently described by our laboratory (Akhmedov et al., 2013, Journal of Visualized Experiments, Accepted). Briefly, prior to dissection, animals were anesthetized by injection of isotonic MgCl_2_ (337 mM) at a volume of 50%–60% of their body weight. After a 5–10 minute incubation, abdominal ganglion was removed by surgical operation and subjected to 0.1% protease treatment for 30 minutes at 34.5°C followed by ASW perfusion (flow rate: 150 μl/min) at room temperature. Ganglion was desheathed under a binocular stereomicroscope followed by a wash with 100% ethanol.

Selected identified neurons (R15, L7, L11 and R2) were recognized based on their characteristic position in abdominal ganglia and isolated using glass microelectrodes, and placed in Trizol. RNA was isolated according to the standard Trizol (Invitrogen) protocol and RNA concentration was determined spectrophotometrically on a nanodrop at 230, 260 and 280 nm. The total RNA from single neurons were subjected to two rounds of linear T7 RNA Polymerase–driven transcription using MessageAmp™ II aRNA Amplification Kit from Ambion.

### Microarray and bioinformatics analysis

A custom designed microarray [36] was used in gene expression experiments. Microarray analysis was carried out in collaboration with the genomics core at Sanford-Burnham Medical Research Institute, Orlando, Florida. RNA from three animals was used in each group (mature and old) for the analysis. The purity and integrity of the total RNA were analyzed on RNA Nano chip (Agilent Technologies) using Eukaryote Total RNA Nano series protocol. The total RNA was subjected to two rounds of linear IVT-amplification and labeled with Cy3-labeled CTP using Amino Allyl MessageAmp II Amplification kits (Ambion). The resulting Cy3 dye incorporated aRNA was quantified using ND-1000 spectrophotometer (Nano Dro Technologies) and 1.65 ug of labeled aRNA was hybridized on to Agilent’s 4 × 44 k format arrays (Agilent Technologies). After hybridization, the arrays were washed following the manufacturer’s protocol using Gene Expression Wash Pack (Agilent Technologies) and scanned using the Agilent C Scanner. The intensities of the scanned fluorescence images were extracted with Agilent Feature Extraction software version 10.7.3.1. The data reported in this paper have been deposited in the Gene Expression Omnibus (GEO) database (http://www.ncbi.nlm.nih.gov/geo) with the accession number GEO: GSE46618.

The mean signals were background corrected and transformed to the log2 scale. The data was then normalized between arrays by quantile approach. The empirical Bayes moderated t-statistics, which is implemented in the limma Bioconductor package [83], were used for differential expression detection. Genes with at least 2-fold changes and p-value 0.05 were considered as significant. The hierarchical clustering and other statistical analyses were performed using R/Bioconductor [84]. Principal component analysis (PCA) was performed with Partek Genomics Suite (Partek Inc. St. Louis, MO).

To ensure maximum accuracy for annotation of the signature list (1083 microarray probes), the probes were blasted against the assembled RNA transcripts (http://www.aplysiagenetools.org). First, a BLAST database was prepared. The resulting database was verified to contain the expected 1253635 sequences. Then, a BLASTN was performed against this database using the flat file containing the microarray probes. We chose the "tabular" output format to ensure easy manipulation programmatically.

The method described above produced 737 transcripts that matched the microarray probes (E-value < 1). Out of 737 transcripts, 572 were successfully blasted (tblastx) against nr database (E-value < 10) using the BLAST2GO application (http://www.blast2go.com). 247 transcripts were upregulated and 325 were downregulated in R15 mature versus R15 old neurons and these transcripts were studied by IPA analysis. To annotate the above 737 transcripts based on Gene Ontologies, the transcripts were blasted (blastx) against nr database (E-value < 1.0E-3). Mapping function in Blast2GO links different protein IDs in the BLAST hits to Gene Ontology database (GO consortium <http://www.geneontology.org>). The GO database contains several million functionally annotated genes for hundreds of species from public resources provided by the NCBI, PIR and GO. To ensure high quality annotation, only ontologies obtained from hits with E-value < 1.0E-6 are used for annotation and shown in the GO graphs. Annotation cutoff was set to 55 and GO weight was set to 5. Filter GO by taxonomy parameter was set to none.

Mapping and annotation was performed based on default parameters in the BLATS2GO application. Additionally, InterProScan function was utilized to improve the gene ontology analysis results. GO analysis for upregulated and downregulated hits shown in the Additional file 1 was performed using GO-Slim and combined graph function in BLAST2GO. The sequence filter was set to 5.

### Ingenuity Pathway Analysis (IPA)

The data mining software, Ingenuity Pathway Analysis (IPA), was used for further analysis of the gene list obtained from the BLATS2GO analysis. The top blast hits accessions exported from Blast2GO were converted to Entrez Gene IDs using NCBI Batch Entrez tool, and then imported into IPA. IPA defaults to human however does have mouse and rat genes within it. Only if a human gene ortholog is not available, the mouse gene will be mapped. In our analysis, 190 human genes were mapped to the above gene list in IPA. This list was used for functional and network analysis using the Core Analysis feature in IPA. Red indicates upregulation and green indicates downregulation in R15 mature neurons relative to R15 old neurons.

### Quantitative real time PCR (qPCR)

The genes identified by microarray analysis were validated by qPCR. Total RNA was isolated from single neurons as described earlier and was subjected to two rounds of linear IVT-amplification. 1 μg of RNA was used in 40 ul of reaction performed with qScript™ cDNA SuperMix from Quanta BioSciences according to the manufacturer's protocol. All amplifications were primed by pairs of chemically synthesized 18- to 24- mer oligonucleotides designed using freely available primer design software (Primer-3,) to generate target amplicons of 70–110 bp. Sequence information of the forward and reverse primers used are listed in Additional file 2: Table S5. The qPCR mixture had a total volume of 10 μl containing 2 μl of H2O, 2 μl of cDNA, 5 μl of 2X Master Mix, 1.0 μl of 10 μM (each) forward and reverse primer. The reaction was carried out in a 7900HT Fast Real-Time PCR System (Applied Biosystems Carlsbad, CA) under the following conditions: 95°C for 10 minutes, followed by 40 cycles of 95°C for 15 seconds, 60°C for 1 minute. There were five biological replicates and four technical replicates for each biological replicate. Quantification of the target transcripts was normalized to the *Aplysia*18S reference gene using the Pfaffl method [85]. Student t test was used to detect genes with statistically significant expression levels between mature and old single neurons where *P-value < 0.05, **P-value < 0.01, ***P-value < 0.001.

### Availability of supporting data

All the microarray data is deposited at Gene Expression Omnibus (GEO) database (http://www.ncbi.nlm.nih.gov/geo) with the accession number GEO: GSE46618. All the other supporting data (Additional file 1 and Additional file 2) are deposited at LabArchives (http://www.labarchives.com/) in the folder named Puthanveettil-BMC Genomics).

## Abbreviations

ASW: Artificial Sea Water; BLAST: Basic Local Alignment Search Tool; CNS: Central nervous system; CREB: cAMP Response Element-Binding protein; GEO: Gene Expression Omnibus; GO: Gene ontology; IVT: *In Vitro* Transcription; PCA: Principal component analysis; qRTPCR: Quantitative real time PCR; S6K: S6 kinase.

## Competing interests

The authors declare that they have no competing interests.

## Authors’ contributions

SP, BK and KA conceived of the study. TC generated aged animals. BK, MS and SP wrote the article. MS & AC analyzed the microarray data and conducted GO and IPA analysis. All authors have read and approved the manuscript for publication.

## Supplementary Material

Additional file 1 Figure S1 Relative expression of CREB1 in mature and old abdominal ganglia. CREB1 mRNA levels in the mature and old abdominal ganglia were quantified and normalized to 18S rRNA internal standard. CREB1 do not show any significant change in expression with aging in the whole abdominal ganglia. The error bars indicate standard error of the mean (SEM). Student’s t test determined statistical significance where *P*-value < 0.05 was considered significant. **Figure S2.** Statistical analysis of microarray data. (A) Scatter plots and correlation coefficients for each microarray experiment pair R15 mature and old single neurons. Mature #2 Rep1 and Old #3 Rep1 represent the replicates within each slide (internal replicates). (B) Principle Component Analysis (PCA) mapped scatter plot of technical replicates: The global gene expression profiles of the R15 neurons from both mature and old *Aplysia* were analyzed by PCA. The figure represented the first two principal components of microarray analysis data (PC1, PC2 and PC3) in X, Y and Z respectively. (Mature Red # 1–3, Old blue# 1–3). **Figure S3.** Identification of biological network functions by Ingenuity Pathway Analysis. A) Network 2: Top functions of the genes were related to cell death and survival, cellular function maintenance and embryonic development. (B) Network 3: This network has 15 genes differentially expressed and the biological functions associated with network 3 are neurological diseases, developmental and hereditary disorders. Node and edge symbols are shown along with network diagram. Red shows genes that are upregulated and green indicates downregulation of the genes. Uncolored notes represent genes that were not identified in our data set as differentially expressed in our analysis, but relevant to this network. Click here for file

Additional file 2 Table S1 Differentially expressed probe list from the microarray analysis of mature and old single R15 neuron RNA. **Table S2.** List of annotated *Aplysia* genes from RNA seq analysis. **Table S3.** Gene Ontology classification of differentially expressed genes from mature R15 neuron. **TableS4.** Ingenuity Pathway Analysis (IPA) of differentially expressed genes from mature R15 neuron. **Table S5.** Primer sequences used for qPCR analysis. Click here for file
