# Supplementary figures and images for: Age-associated bidirectional modulation of gene expression in single identified R15 neuron of Aplysia
Source: BMC Genomics. 2013 Dec 14;14:880. doi: 10.1186/1471-2164-14-880 (PMC3909179; doi:10.1186/1471-2164-14-880)

## Slide 1
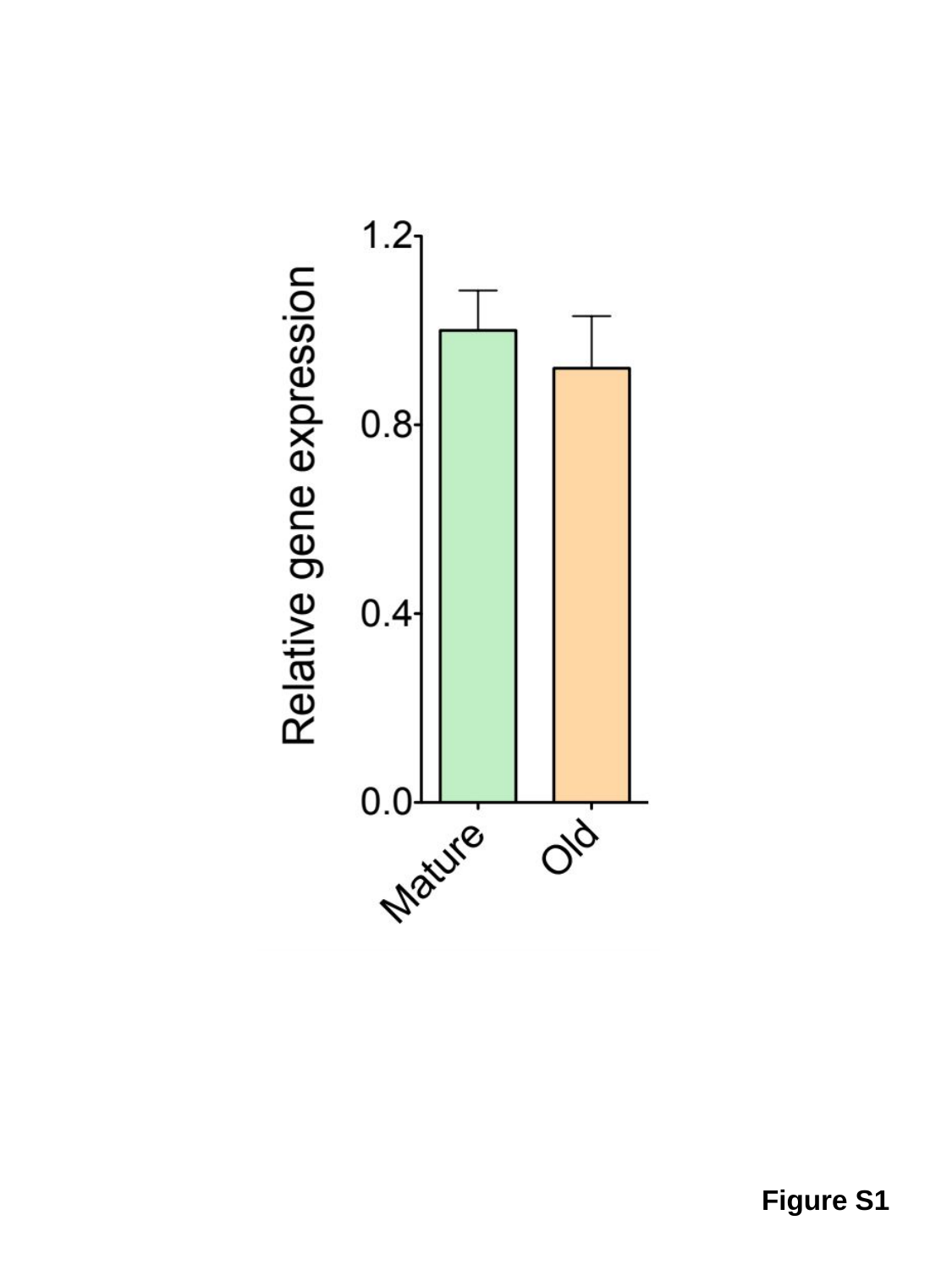

Figure S1

## Slide 2
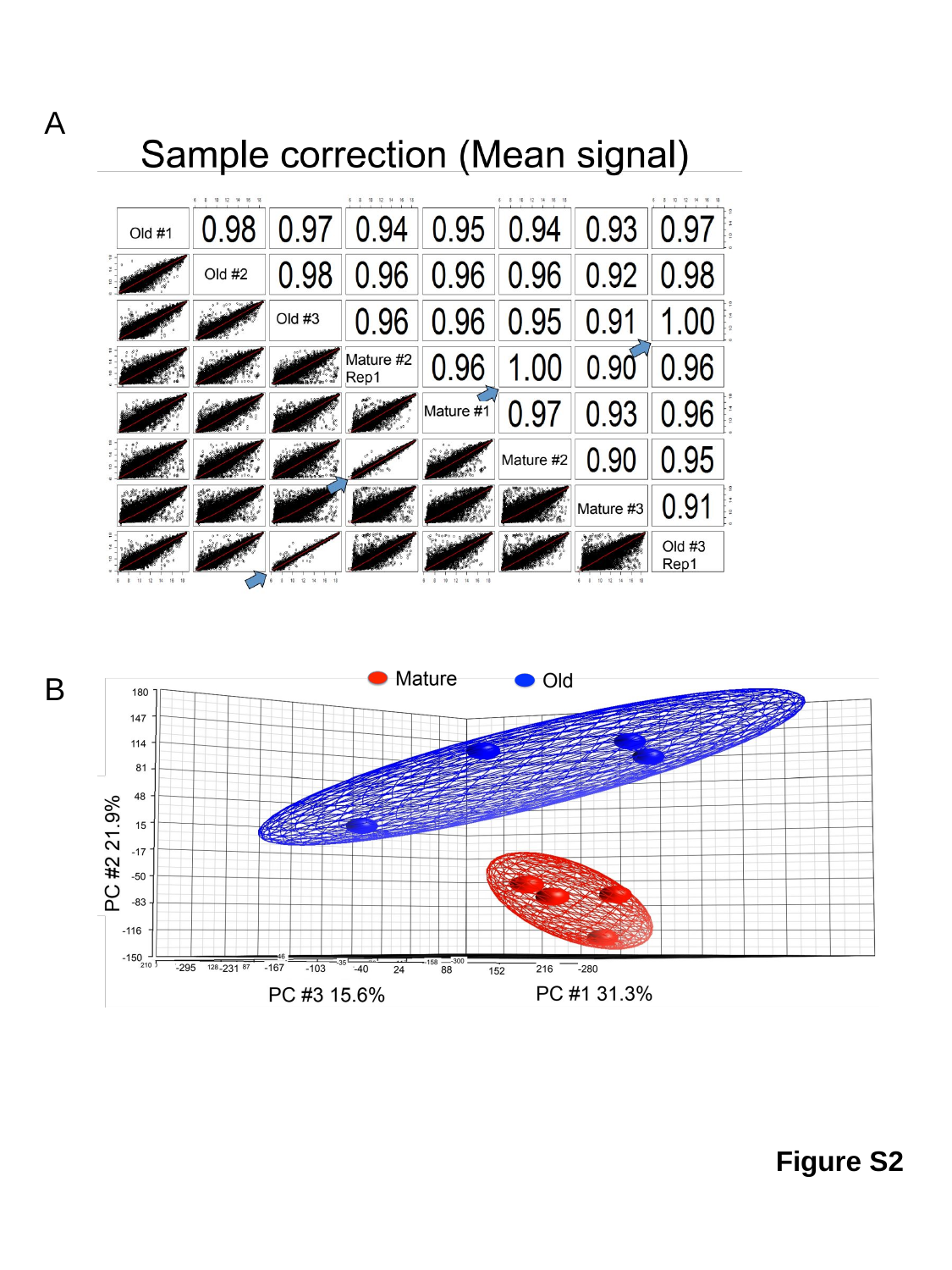

A
B
Figure S2

## Slide 3
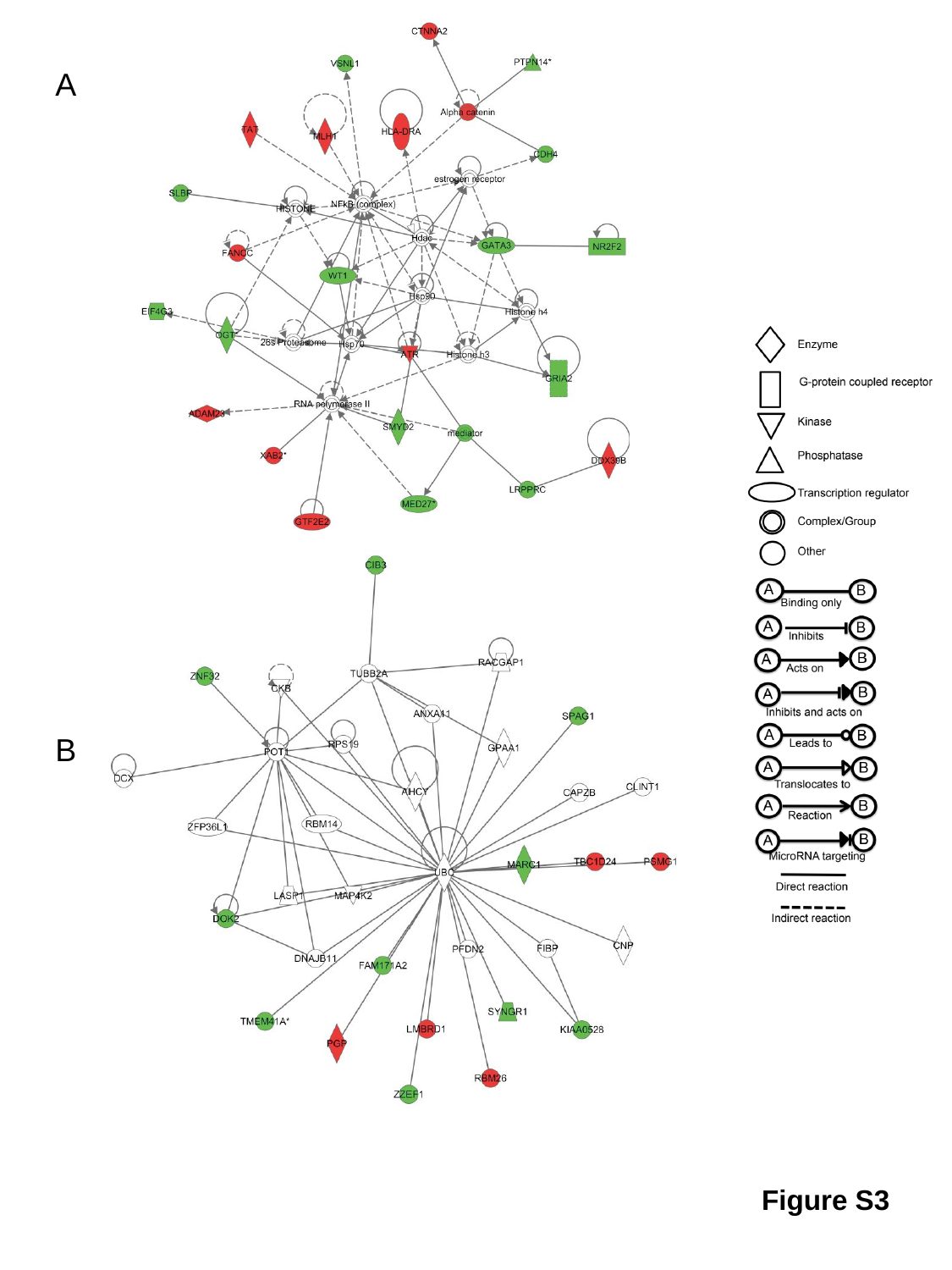

A
B
Figure S3

Supplement: Additional file 1 Figure S1 — Relative expression of CREB1 in mature and old abdominal ganglia. CREB1 mRNA levels in the mature and old abdominal ganglia were quantified and normalized to 18S rRNA internal standard. CREB1 do not show any significant change in expression with aging in the whole abdominal ganglia. The error bars indicate standard error of the mean (SEM). Student’s t test determined statistical significance where P-value < 0.05 was considered significant. Figure S2. Statistical analysis of microarray data. (A) Scatter plots and correlation coefficients for each microarray experiment pair R15 mature and old single neurons. Mature #2 Rep1 and Old #3 Rep1 represent the replicates within each slide (internal replicates). (B) Principle Component Analysis (PCA) mapped scatter plot of technical replicates: The global gene expression profiles of the R15 neurons from both mature and old Aplysia were analyzed by PCA. The figure represented the first two principal components of microarray analysis data (PC1, PC2 and PC3) in X, Y and Z respectively. (Mature Red # 1–3, Old blue# 1–3). Figure S3. Identification of biological network functions by Ingenuity Pathway Analysis. A) Network 2: Top functions of the genes were related to cell death and survival, cellular function maintenance and embryonic development. (B) Network 3: This network has 15 genes differentially expressed and the biological functions associated with network 3 are neurological diseases, developmental and hereditary disorders. Node and edge symbols are shown along with network diagram. Red shows genes that are upregulated and green indicates downregulation of the genes. Uncolored notes represent genes that were not identified in our data set as differentially expressed in our analysis, but relevant to this network. [file 1471-2164-14-880-S2.pptx]
